# Anatomical Characteristics of Pulmonary Veins for the Prediction of Postoperative Recurrence after Radiofrequency Catheter Ablation of Atrial Fibrillation

**DOI:** 10.1371/journal.pone.0093817

**Published:** 2014-04-04

**Authors:** Wei Wei, Jun-Bo Ge, Yu Zou, Li Lin, Ying Cai, Xue-Bo Liu, Wen-Qing Zhu

**Affiliations:** 1 Department of Cardiovascular Medicine, East Hospital, Tongji University School of Medicine, Shanghai, China; 2 Department of Cardiovascular Medicine, Zhongshan Hospital, Fudan University, Shanghai, China; Georgia Regents University, United States of America

## Abstract

**Background:**

The relationship between focal pulmonary vein potential and atrial fibrillation (AF) has been confirmed. Pulmonary vein (PV) isolation and circumferential pulmonary vein ablation have been the most commonly used procedures of radiofrequency ablation. However, few studies have investigated the relationship between anatomical characteristics of PV and AF recurrences after radiofrequency ablation.

**Methodology:**

For 267 AF patients treated by radiofrequency catheter ablation, the anatomic structure characteristics of pulmonary veins were assessed by multi-slice spiral computed tomography while the values of left atrial diameter (LAD) were measured with transesophageal ultrasonic cardiogram. After radiofrequency catheter ablation, postoperative recurrence was evaluated during a 10-month term follow-up.

**Principal Findings:**

During follow-up, postoperative recurrence occurred in 44 patients. The mean diameters of LAD, left superior PV, right superior PV, all left PV, and all superior PV were significantly larger in patients with postoperative recurrence (Recurrence vs. Non-recurrence group; 43.9 ± 6.4 mm vs. 40.7 ± 5.6 mm; 18.4 ± 2.1 mm vs. 17.1 ± 3.1 mm; 18.2 ± 2.8 mm vs. 17.2 mm ± 3.9 mm; 16.4 ± 1.5 mm vs. 15.6 ± 2.5 mm; 18.3 ± 2.1 mm vs. 17.1 ± 3.0 mm; respectively; all *P* < 0.05). Multivariable survival analysis showed that the type and the course of AF, LAD, and the diameters of all superior PV were the independent risk factors for the postoperative recurrence after radiofrequency catheter ablation.

**Conclusions:**

The enlargements of all superior PV and LAD, long course of diseases, and persistent AF were the independent risk factors for the postoperative recurrence after radiofrequency catheter ablation.

## Introduction

Atrial fibrillation (AF) is one of the most commonly observed clinical tachyarrhythmias. In recent years, in addition to drugs, radiofrequency ablation for the treatment of AF has undergone rapid development and has become one of the most important procedures for AF [1]. However, globally, considerable variations in the success rates of radiofrequency ablation for AF have been reported, which may be related to the differences in procedures, population characteristics, and evaluation indexes. In order to better evaluate the results of AF radiofrequency ablation procedures, in the last few years, several studies have assessed numerous factors that may be related to recurrence after radiofrequency ablation, such as age, left atrial size, organic heart disease, type of AF, and duration of AF [2], [3]. These important factors might influence the efficacy of radiofrequency ablation but still require further confirmation from large-scale studies.

The relationship between focal pulmonary vein potential and AF has been confirmed in previous studies. Currently, the most commonly used radiofrequency ablation procedures are pulmonary vein isolation and circumferential pulmonary vein ablation [3]. However, few studies have investigated the relationship between anatomical characteristics of the pulmonary vein and AF recurrences after radiofrequency ablation.

In this study, transesophageal ultrasonic cardiography was used to measure the left atrial diameter, and multi-slice spiral computed tomography (CT) was used to measure CT values and variations in the pulmonary veins of AF patients who had undergone surgery. These two measures combined with other indicators were used to evaluate the factors related to postoperative recurrences of AF. In addition, we analyzed the relationship between anatomical characteristics of the pulmonary vein and postoperative AF recurrence.

## Materials and Methods

### Research subjects

With the approval of the institutional review board of Zhongshan Hospital, Fudan University, a total of 267 patients with atrial fibrillation who had undergone radiofrequency catheter ablation between January 2007 and May 2009 were retrospectively enrolled. Written informed consent was obtained from the patients before operation. All patients had undergone preoperative transesophageal ultrasonic cardiography to exclude patients with valvular heart disease, congenital heart diseases, left atrial thrombosis, or other organic heart diseases. The left atrial diameter had been recorded. All patients had undergone pulmonary vein CT angiography (scanning range from 1 cm above the carina to the apex of the heart) using 128-row multi-slice spiral CT three days prior to radiofrequency ablation. Using CT scan, the diameters of the left superior pulmonary vein, the left inferior pulmonary vein, the right superior pulmonary vein, and the right inferior pulmonary vein were measured, and other variations of pulmonary veins were also recorded. In addition, the overall mean diameters of the left, right, superior, and inferior pulmonary veins were calculated (the overall mean of the left pulmonary vein: the mean value of the left superior pulmonary vein diameter and the left inferior pulmonary vein diameter; the other overall means were calculated using the same method).

### Surgical procedure

The left subclavian vein and both the femoral veins of all patients (including those with paroxysmal and persistent AF) were punctured under local anesthesia (2% lidocaine), and a 12-lead coronary sinus electrode was placed. A dose of 5000–6000 U heparin was injected through a catheter, and a dose of 1000 U of heparin was added every hour during the operation. Heparin was diluted in physiological saline and was infused continuously to prevent thrombosis. Electroanatomical imaging of the left atrium and pulmonary veins was completed under the guidance of a three-dimensional mapping system for all patients, and ablation was performed based on the electroanatomical imaging obtained. The ablation energy was provided by a saline-irrigated ablation catheter, and the ablation temperature was set to be lower than 43°C. Circumferential pulmonary vein ablation was employed under the guidance of the Carto system. The pulmonary vein ablation site was located in the pulmonary vein antrum, which is 0.5–1.0 cm outside the pulmonary vein opening. The default power was set at 35–40 W (reduced to 30 W for the posterior wall), temperature was at 43°C, saline flow rate was 17 mL/min (flow rate between ablation: 2 mL/min), and the discharge at each point lasted for 20–30 s. After circumferential ablation was completed, the lasso method was used to measure pulmonary vein potential for verifying blockade by the ablation circumference, and points that were not ablated were supplemented to achieve pulmonary vein isolation. The ablation end-point was complete pulmonary vein isolation. If cardioversion was not achieved, electrical cardioversion was administered, and the patient was observed for 15 min. If cardioversion was still not achieved, then additional circumferential ablation (left atrial isthmus, left atrium, right atrial isthmus, between the left and right pulmonary vein ablation circumference, etc.) or segmental ablation was performed until either AF reverted to sinus rhythm or when segmented potential disappeared.

### Follow-up

All patients underwent outpatient and telephonic follow-ups. The day of the operation was taken as the starting point of follow-up, and if postoperative recurrence occurred, the procedure was considered ineffective. The end-point of the follow-up was 300 days after the operation. Outpatient follow-up was performed at 1, 3, and 6 months after the operation. The analysis at the follow-up included the patient's symptoms and 12-lead ECG and 24-h Holter findings. If the Holter examination showed atrial tachyarrhythmias (atrial fibrillation, atrial flutter, or atrial tachycardia), the incident was viewed as a recurrence. In the event of recurrence, the frequency and duration of atrial tachyarrhythmias were investigated. All patients were followed-up for at least 6 months and received telephone follow-up after 300 days. Telephonic follow-up included questioning to determine symptoms of recurrence, time of recurrence, frequency and duration of the attack, any drug reactions, etc.

### Statistical analysis

All data were processed using SPSS 17.0 software. Measurement data were expressed in the form of mean ± standard deviation (x ± s). Categorical data were expressed in terms of frequency. Comparisons of the two samples were performed using independent sample *t*-test or chi-square test. Univariate and multivariate Cox regression was employed to analyze the relationship between the various factors and recurrence; nominal variables were grouped based on the qualitative categories, discrete ordinal variables based on recognized segmentation values, and continuous variables based on the optimal cutoff obtained by the minimum P-value method obtained by the x-tile software. A *P* value of < 0.05 was considered as statistically significant.

## Results

### Description of basic information

A total of 267 patients (176 male and 91 female) with a mean age of 57.4 ± 10.6 years were included in the study. In all, 57 patients had persistent AF and 142 patients had hypertension, 7 had hyperthyroidism, 12 had coronary heart disease, and 17 had experienced stroke. All patients had been taking oral warfarin for more than a month before the procedure. All patients also had a medical history of taking more than one type of anti-arrhythmia drugs, of which 232 (86.89%) had previously taken amiodarone. All patients continued taking warfarin orally 3 days after the operation for 3 months and amiodarone orally for 1 month. None of the patients reported either drug-related arrhythmia or bleeding during follow-ups.

### Structural features of the pulmonary vein and left atrial diameter

Amongst the 267 patients, 80 (29.96%) showed pulmonary vein abnormalities (Table 1), of which variations in the right middle lobar pulmonary vein and early branching of the pulmonary vein had the highest frequency that occurred in 35 patients each, accounting for 43.75% of all patients with variations. In addition, 7 patients (8.75%) had shared pulmonary vein branches, as well as 3 patients (3.75%) had independent pulmonary vein, pulmonary vein stenosis, and other variations. In all, 224 patients (83.90%) had 4 pulmonary veins, while 6 patients had 3 pulmonary veins, 35 patients had 5 and 2 patients had 6.

**Table 1:** Structural features of the pulmonary vein and left atrial diameter.

Variables	PV variation, n	LSPV, mm	LIPV, mm	RSPV, mm	RIPV, mm	OLPV, mm	ORPV, mm	OSPV, mm	OIPV, mm	LAD, mm
Overall	80	17.3±3.0	14.2±3.0	17.3±3.7	15.2±3.1	15.8±2.4	16.3±2.9	17.3±2.9	14.7±2.6	41.2±5.9
Non-recurrence group	68	17.1±3.1	14.1±3.1	17.2±3.9	15.1±3.0	15.6±2.5	16.1±2.9	17.1±3.0	14.6±2.5	40.7±5.6
Recurrence group	12	18.4±2.1	14.5±2.1	18.2±2.8	15.7±3.8	16.4±1.5	17.0±3.0	18.3±2.1	15.1±2.5	43.9±6.4
P value (Non-recurrence vs Recurrence group)	0.670	0.001	0.369	0.030	0.235	0.005	0.077	0.002	0.250	0.003

NOTE: PV, pulmonary vein; LSPV, left superior pulmonary vein; LIPV, left inferior pulmonary vein; RSPV, right superior pulmonary vein; RIPV, right inferior pulmonary vein; OLPV, overall left pulmonary vein; ORPV, overall right pulmonary vein; OSPV, overall superior pulmonary vein; OIPV, overall inferior pulmonary vein; LAD, left atrial diameter.

After 300 days of follow-up, of the 267 patients, 44 (16.5%) showed recurrence, of which 21 showed atrial flutter, 18 showed AF, and 5 showed atrial tachycardia. A comparison between the recurrence and non-recurrence groups revealed that the mean diameters of the left atrium, left superior pulmonary vein, and right superior pulmonary vein and the overall mean left pulmonary vein diameters and the overall superior pulmonary vein diameters showed significant increase (*P* < 0.05), while the mean diameters of the left inferior pulmonary vein, right inferior pulmonary vein, right pulmonary veins, and inferior pulmonary veins, as well as the number of people with pulmonary vein variations were not statistically different between the two groups (Table 1).

Therefore, the anatomical structures of the pulmonary veins and the left atrial diameter might be closely related to recurrence after radiofrequency catheter ablation for AF.

### Analysis of factors related to recurrence of AF after radiofrequency catheter ablation

#### Selection of cutoff values

Gender, type of AF, and the presence of pulmonary vein variations were the nominal variables and were grouped based on their qualitative categories. Age and disease duration were discrete ordinal variables and were grouped based on recognized standards and clinical experience. Age was categorized into three groups: <40 years, 40–60 years, and >60 years. Disease duration was grouped into <5 years, 5–10 years, and >10 years.

The diameter of each pulmonary vein opening and the left atrial diameter were continuous variables, and the minimum *P* value method was used to select their cutoff values, by selecting the smallest *P* value amongst all *P* value < 0.05 as the optimal cutoff value. This was calculated using X-tile software [4], and the optimal cut-off values obtained were: 16.1 mm for the left superior pulmonary vein, 14.0 mm for the left inferior pulmonary vein, 16.2 mm for the right superior pulmonary vein, 17.8 mm for the right inferior pulmonary vein, 46 mm for the left atrial diameter, 14.6 mm for all the left pulmonary veins, 15.4 mm for all the right pulmonary veins, 16.4 mm for all the superior pulmonary veins, and 13.8 mm for all the inferior pulmonary veins.

#### Multivariate Cox regression analysis of the variables and postoperative recurrence

As observed in Table 2, the variables were grouped according to the abovementioned cutoff values, and the end-point for follow-up observation was 300 days after catheter ablation. Cox univariate analysis revealed that risk factors for possible AF postoperative recurrence included: opening diameters of all pulmonary veins, overall mean diameters of pulmonary vein opening on all sides, left atrial diameter, AF type, and AF duration. Postoperative recurrence was not significantly related to presence of hypertension, coronary heart diseases, or hyperthyroidism.

**Table 2:** Univariate and multivariate Cox regression analysis of factors associated with postoperative recurrence (stepwise method).

Variables	Univariate analysis			Multivariate analysis	
	HR (95% CI)	P		HR (95% CI)	P
Gender	NA	NS		NA	NA
Age	NA	NS		NA	NA
Hypertension	NA	NS		NA	NA
Coronary heart disease	NA	NS		NA	NA
Hyperthyroidism	NA	NS		NA	NA
AF type	3.154 (1.736–5.729)	0.000		3.589 (1.925–6.690)	0.000
AF duration	1.807 (1.257–2.599)	0.001		2.084(1.449–2.996)	0.000
LAD	5.177(2.861–9.369)	0.000		4.575 (2.504–8.360)	0.000
PV variation, n	NA	NS		NA	NA
LSPV	4.058 (1.599–10.297)	0.003		NA	NS
LIPV	3.302 (1.631–6.685)	0.001		NA	NS
RSPV	2.812 (1.307–6.051)	0.008		NA	NS
RIPV	1.850 (0.992–3.451)	0.053		NA	NA
OLPV	3.458 (1.363–8.773)	0.009		NA	NS
ORPV	1.772(0.913–3.440)	0.091		NA	NA
OSPV	3.980 (1.682–9.417)	0.002		3.496 (1.368–8.938)	0.009
OIPV	1.787 (0.903–3.537)	0.095		NA	NA

Note: Multivariate analysis was performed using the Cox multivariate proportional hazard regression model with a stepwise method (forward, likelihood ratio); NA, not assessed; NS, not significant; AF, atrial fibrillation; PV, pulmonary vein; LSPV, left superior pulmonary vein; LIPV, left inferior pulmonary vein; RSPV, right superior pulmonary vein; RIPV, right inferior pulmonary vein; OLPV, overall left pulmonary vein; ORPV, overall right pulmonary vein; OSPV, overall superior pulmonary vein; OIPV, overall inferior pulmonary vein; LAD, left atrial diameter.

In order to reduce errors caused by individual factors, we established three Cox multivariate regression models using the Enter mode, which independently incorporated the opening diameters of all pulmonary veins, the overall mean opening diameters of left and right pulmonary veins, and the overall mean opening diameters of the inferior and superior pulmonary veins. We found that left atrial diameter, left superior pulmonary vein diameter, overall mean left pulmonary vein diameters, overall mean superior pulmonary vein diameters, AF type, and AF duration were independent risk factors for postoperative recurrence of AF (Table 3).

**Table 3:** Multivariate Cox regression analysis of factors associated with postoperative recurrence (enter method).

Variables	A		B		C	
	HR (95% CI)	P	HR (95% CI)	P	HR (95% CI)	P
AF type	3.207 (1.700–6.047)	0.000	3.416 (1.812–6.437)	0.000	3.572 (1.905–6.696)	0.000
AF duration	1.985(1.367–2.881)	0.000	2.148 (1.497–3.081)	0.000	2.035(1.407–2.945)	0.000
LAD	4.302 (2.341–7.904)	0.000	4.619 (2.531–8.429)	0.000	4.374 (2.392–7.996)	0.000
LSPV	3.107 (1.124–8.585)	0.029				
LIPV	NA	NS				
RSPV	NA	NS				
OLPV			2.811 (1.089–7.257)	0.033		
OSPV					2.728 (1.133–6.573)	0.025

Note: Multivariate analysis was performed using the Cox multivariate proportional hazard regression model with an enter method; NA, not assessed; NS, not significant; AF, atrial fibrillation; LSPV, left superior pulmonary vein; LIPV, left inferior pulmonary vein; RSPV, right superior pulmonary vein; OLPV, overall left pulmonary vein; OSPV, overall superior pulmonary vein; LAD, left atrial diameter.

Factors in the univariate analysis, which were found to be possibly related to recurrence, were incorporated stepwise to establish a multivariate Cox regression model. It was found that left atrial diameter, left superior pulmonary vein diameter, AF type, and AF duration were independent risk factors for postoperative recurrence while the overall mean left pulmonary vein diameter was not (Table 2). Therefore, larger left atrial diameter, increased mean width of the inferior pulmonary vein, persistent AF, and AF duration of more than 10 years were independent risk factors for postoperative recurrence.

## Discussion

After more than two centuries of debate, we are currently approaching a consensus: the occurrence and maintenance of AF require certain triggering factors (the point of origin on the pulmonary vein) and mechanisms for maintaining AF (electrical and structural remodeling of the left atrium). Hence, it is important to thoroughly transform the inherent characteristics and mutual relationships of the pulmonary vein and the left atrium in order to prevent and treat AF, thereby destroying the pre-existing environment, which was susceptible to AF. Until the 1990s, Haissaguerre et al [5] conducted numerous studies and proposed that the pulmonary vein is an important position in the triggering mechanism of AF, thus creating the new method of radiofrequency catheter ablation for AF. Even though radiofrequency catheter ablation has shown good efficacy for AF, the high rate of postoperative recurrence is one of the main factors limiting its widespread use [6]. Based on this, our study emphasized the evaluation of the relationship between pulmonary vein structural characteristics as well as left atrial diameter and AF recurrence after radiofrequency ablation. This will lay down the theoretical foundations for the selection of surgical indications for AF as well as the prevention and treatment of postoperative recurrence.

Previous research has shown that the structural and electrical remodeling of the left atrium is an important mechanism for the occurrence and maintenance of AF [7]. After the enlargement of the atrium, maintaining normal electrical activity will be difficult; this maintenance will cause corresponding changes to the automaticity of the atrial muscles, which will increase their susceptibility to AF. The enlargement of the left atrium will cause continuous remodeling of the atrium, which will lead to increased susceptibility to atrial reentry. This will disrupt normal conduction pathways and reduce the efficacy of radiofrequency ablation and the success rate of operations. Moreover, several studies have also shown that the enlargement of left atrium is an independent risk factor for postoperative recurrence of AF [3], [8], [9]. In this study, we found via multivariate analysis that enlargement of the left atrium is an independent risk factor for postoperative recurrence of AF, thereby verifying that left atrial enlargement plays an important role in the occurrence, maintenance, and postoperative recurrence of AF.

Previous studies on the relationship between the anatomical structure of the pulmonary veins and the occurrence and development of AF have mainly focused on anatomical variations of pulmonary veins [10], [11]. In this study, the rate of anatomical variations in the pulmonary veins of the patients was 29.96%. Multivariate regression analysis did not indicate that pulmonary vein variation was an independent risk factor for postoperative recurrence. Further investigation is still needed to understand the relationship between pulmonary vein anatomical variations and the occurrence and development of AF.

This study focused on evaluating the relationship between diameters of opening of the pulmonary veins and recurrence of AF after radiofrequency ablation. There are no similar studies to date. We found that when compared to the non-recurrence group, the recurrence group had significant increases in the mean diameters of the left superior, right superior pulmonary veins and the overall means of the left and superior pulmonary veins, which indicate that the opening diameters of pulmonary veins might be related to postoperative recurrence. Through multivariate analysis, we further confirmed widening of the superior pulmonary vein is an independent risk factor for determining whether postoperative recurrence will occur. Past studies have already demonstrated that the heterotopic triggering point for AF, that is, the heterotopic starting point on the pulmonary vein, is usually located on the superior pulmonary vein [5]. The significant widening of the superior pulmonary vein could result in the following: (1) An increase in electrical activity at the AF starting point, which will increase the difficulty and complexity and reduce the success rate of the operation. (2) An absence of a clear definition of the ablation line and the end-point due to the current limitations in ablation mapping and techniques; hence, individualized treatment is lacking, and for those with enlarged pulmonary veins, the ablation line will increase, resulting in increased probability of ablation gaps, which will lead to postoperative recurrence. (3) Deep ablation points that would increase diameter of the pulmonary veins, leading to the recovery of electrical conduction. (4) Atrial electrical remodeling in those with increased diameters pulmonary veins, in which AF cannot be fully treated using only pulmonary vein isolation. (5) Enlargement of the pulmonary vein that might cause the formation of electrical connections between the pulmonary veins that can easily lead to the emergence of a trigger point beyond the pulmonary veins.

Our study also found that persistent AF and AF duration are independent risk factors for postoperative recurrence. With the increase of AF duration, regardless of whether the disease duration is longer or persistent, significant structural and electrical remodeling would have occurred due to repeated stimulations of the left atrium and pulmonary veins [3], [8], [9], [12]. This would change the sensitivity of atrial muscles to radiofrequency ablation energy and hence, adversely affect the success rate of the operation.

In summary, left atrial diameter is closely related to the development and occurrence of AF, while pulmonary vein structural characteristics might also be closely related to postoperative recurrence of AF; however, further investigation is still required in this regard. During clinical work-up of patients for this procedure, attention needs to be paid to the left atrial diameter and structural characteristics of pulmonary veins, as this might help us to better select the ideal treatment modality and assess the patient's prognosis.
